# OCC-Based Positioning Method for Autonomous UAV Navigation in GNSS-Denied Environments: An Offshore Wind Farm Simulation Study

**DOI:** 10.3390/s25247569

**Published:** 2025-12-12

**Authors:** Ju-Hyun Kim, Sung-Yoon Jung

**Affiliations:** Department of Electronic Engineering, Yeungnam University, Gyeongsan 38541, Republic of Korea; lpyeongl00@gmail.com

**Keywords:** offshore wind turbine, optical camera communication (OCC), perspective-n-points (PnP), uncrewed aerial vehicle (UAV), vehicle positioning, UAV localization, vehicle-to-infrastructure (V2I), GNSS-denied environment, YOLOv8

## Abstract

Precise positioning is critical for autonomous uncrewed aerial vehicle (UAV) navigation, especially in GNSS-denied environments where radio-based signals are unreliable. This study presents an optical camera communication (OCC)-based positioning method that enables real-time 3D coordinate estimation using aviation obstruction light-emitting diodes (LEDs) as optical transmitters and a UAV-mounted camera as the receiver. In the proposed system, absolute positional identifiers are encoded into color-shift-keying-modulated optical signals emitted by fixed LEDs and captured by the UAV camera. The UAV’s 3D position is estimated by integrating the decoded LED information with geometric constraints through the Perspective-n-Point algorithm, eliminating the need for satellite or RF-based localization infrastructure. A virtual offshore wind farm, developed in Unreal Engine, was used to experimentally evaluate the feasibility and accuracy of the method. Results demonstrate submeter localization precision over a 50,000 cm flight path, confirming the system’s capability for reliable, real-time positioning. These findings indicate that OCC-based positioning provides a cost-effective and robust alternative for UAV navigation in complex or communication-restricted environments. The offshore wind farm inspection scenario further highlights the method’s potential for industrial operation and maintenance tasks and underscores the promise of integrating optical wireless communication into autonomous UAV systems.

## 1. Introduction

Precise positioning is essential for autonomous uncrewed aerial vehicle (UAV) operations, particularly in GNSS-denied environments where radio-based localization signals become unreliable. Although global positioning systems (GPS) and inertial navigation systems (INS) are widely used for UAV localization, both approaches have inherent limitations. GPS offers high accuracy under ideal conditions but is vulnerable to signal blockage, electromagnetic interference, and multipath reflections in complex industrial or offshore settings. INS is robust against external interference and supports real-time processing, yet it experiences cumulative drift during prolonged operation [1]. These limitations underscore the need for RF-independent, vision-based positioning methods capable of maintaining high precision and reliability in GNSS-degraded environments [2].

Optical camera communication (OCC) has emerged as a promising alternative. OCC operates at the physical communication layer and uses light-emitting diodes (LEDs) as transmitters and image sensors or cameras as receivers. By encoding information through variations in light intensity, color, or flicker modulation, OCC enables both data transmission and vision-based spatial awareness. For UAV navigation, fixed LEDs installed on infrastructure can broadcast absolute spatial identifiers that are captured and decoded by an onboard camera, allowing the UAV to estimate its relative position with respect to the LEDs [3]. This light-based method is immune to electromagnetic interference, compatible with existing optoelectronic hardware, and capable of detecting multiple optical sources in a single frame, which makes it highly effective in GNSS-denied or RF-constrained environments. Offshore wind farms serve as an ideal testbed for evaluating OCC-based positioning.

Offshore wind power is a clean and carbon-free energy source that plays a critical role in climate change mitigation and energy security. Its large installation capacity, renewability, and minimal environmental impact have driven increasing global importance [4]. Offshore wind farms typically achieve a capacity factor of 40–50 percent with lower hourly variability than solar power [5], and they offer large-scale deployment opportunities due to fewer land-use constraints and the presence of stable, high wind speeds [6]. However, as offshore wind farms continue to expand, operation and maintenance (O&M) efficiency has become a major challenge. The Global Offshore Wind Workforce Outlook (2024–2028) projects an increase from approximately 10,639 technicians in 2018 to 76,181 by 2028, yet a shortage of more than 33,000 skilled personnel is anticipated [7]. Remote accessibility, unpredictable weather, and significant safety hazards further increase operational risk and complicate workforce retention. Consequently, UAV-based O&M automation has gained strong attention as a means to enhance safety, efficiency, and scalability [8].

UAVs equipped with high-resolution cameras and sensors can inspect turbine components that are difficult or dangerous to access, collecting visual and environmental data for analysis. However, the reliability and stability of UAV-based inspection depend on accurate and consistent positioning. Errors in position estimation may lead to data misinterpretation, communication failures, or collision risks [9]. A high-precision positioning solution that operates independently of satellite or RF signals is therefore essential for safe autonomous UAV navigation in offshore wind environments. OCC presents a compelling pathway to meet this requirement. Aviation obstruction LEDs are already installed on turbine nacelles and towers, and UAVs inherently contain cameras, making OCC cost-efficient and hardware-light [10,11]. OCC operates in the visible spectrum, avoids RF interference, and benefits from wide camera fields of view that enable simultaneous detection of multiple LEDs. Furthermore, geometric analysis between detected LED image coordinates and their known 3D positions allows the UAV to determine its spatial location while avoiding hazardous proximity to turbine blades.

Motivated by these advantages, this study proposes an OCC-based positioning framework for autonomous UAV navigation in GNSS-denied offshore wind farm environments. In the proposed system, LEDs transmit spatial identifiers using color-shift keying (CSK) modulation, and the UAV estimates its 3D position by combining the decoded LED information with geometric constraints through the Perspective-n-Point (PnP) algorithm. To assess the feasibility and robustness of the framework, a virtual offshore wind farm was constructed using the Unreal Engine simulation platform [12]. Each LED transmits a unique 3-bit identification symbol, and when four or more LEDs are simultaneously detected, the UAV computes its complete 3D position through PnP-based geometric computation [13].

## 2. Related Works

Research on OCC-based localization for UAVs can be broadly categorized into two critical areas: the OCC component and the localization method. In terms of OCC-related work, one study [8] proposed a UAV-based system that employs OCC for O&M activities in offshore wind turbine environments. In [14], an OCC-based vehicle-to-vehicle communication system was implemented in an outdoor driving environment, where LEDs served as transmitters on the leading vehicle and an optical image sensor functioned as a receiver on the following vehicle. This system enabled real-time LED detection, data transmission, and video streaming. Likewise, another study [15] designed a security protocol for vehicle platooning using OCC, where modulated taillights of a leading vehicle acted as transmitters and the front camera of the following vehicle served as the receiver. The authors demonstrated that secure OCC-based systems can be realized without hardware modifications.

Regarding OCC-based localization systems, another study [9] presented a framework for estimating the position of moving vehicles in a vehicle-to-infrastructure configuration using OCC. LED streetlights served as transmitters and vehicle-mounted cameras acted as receivers, and vehicle positions were estimated by applying collinearity conditions between the LEDs and their known absolute coordinates. In [11], a visible light communication system was proposed for vehicle localization using roadside LED lights as transmitters and in-vehicle cameras as receivers. Similarly, ref. [16] developed a system that employed vehicle-to-vehicle and vehicle-to-infrastructure visible light communication, where vehicle tail lamps and tunnel-installed LEDs acted as transmitters and vehicle cameras served as receivers. Localization was achieved using LED positional data detected by the image sensors.

In an indoor context, another study [17] demonstrated real-time UAV localization without GPS by extracting 3D–2D correspondences from multiple visual markers captured by a camera and applying the PnP algorithm. In [18], a lightweight YOLOv8-based object detection model was used to recognize objects, and real-time UAV localization was achieved by correlating the detections with predefined 3D coordinates through the PnP algorithm. In both [17,18], the authors emphasized that PnP performance and the accuracy of 3D–2D correspondence extraction are critical to localization accuracy. Consistent with these findings, the present study highlights precise LED detection as a crucial factor in improving localization performance.

## 3. UAV-Based Position Estimation Scenario Using OCC in Offshore Wind Farms

Offshore wind turbines present significant challenges for installation, access, and maintenance, which often results in safety concerns during O&M activities. A previous study [8] proposed an O&M approach in which UAVs periodically patrol offshore wind farms to address these limitations and collect status information from turbines that are difficult to access manually. Building on and expanding the scenario introduced in [8], the present study proposes an OCC-based UAV localization system tailored to offshore wind power environments. A virtual offshore wind farm and UAV flight environment were created using Unreal Engine, and all experiments were conducted within this simulation platform. The virtual environment consists of ten wind turbines arranged in two rows of five, and the UAV flies at regular intervals between the rows while capturing images of the LEDs installed on the turbines.

**Figure 1:**
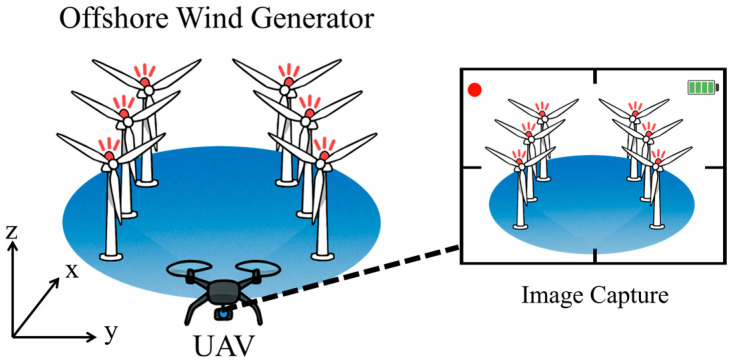
Scenario illustration of the proposed OCC-based UAV positioning experiment in the offshore wind farm environment. The UAV-mounted camera captures frames containing the warning LEDs installed on the wind turbines while flying through the wind farm area.

**Figure 2:**
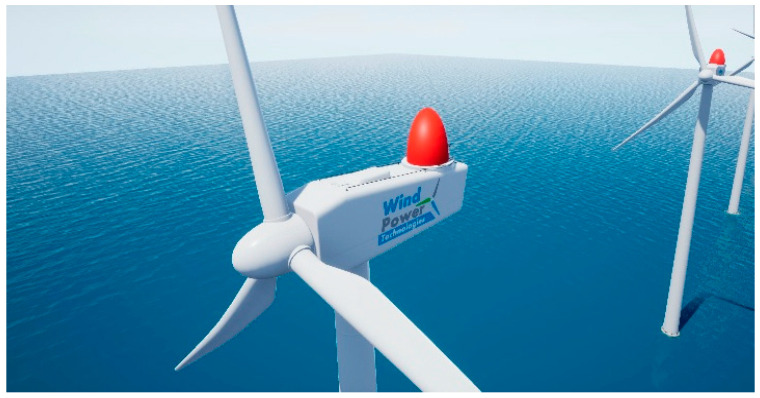
Offshore Wind Turbine & LED Red modeling.

Figure 1 illustrates the overall experimental scenario and shows how the UAV-mounted camera acquires visual information from the warning LEDs installed on the wind turbines during flight. The detailed processing steps for LED detection and PnP-based 3D localization are described in Section 4. Figure 2 depicts the integration of the LED model into the wind turbine model within Unreal Engine.

**Figure 3:**
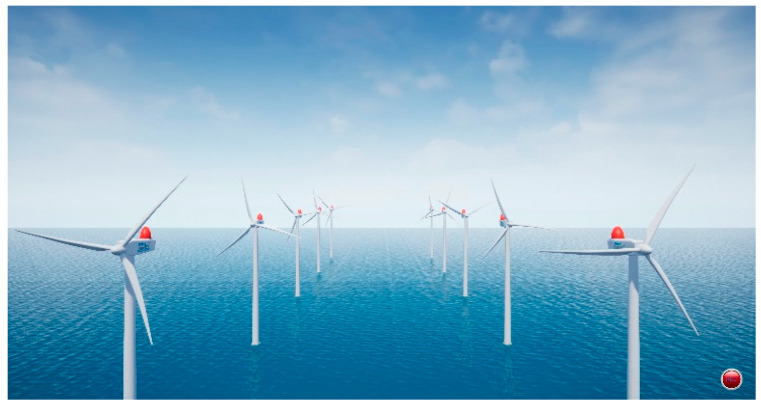
Overview of the virtual offshore wind farm environment implemented in Unreal Engine, captured from a virtual camera positioned at (0, 0, 12,000 cm). The environment comprises ten wind turbines equipped with aviation warning LEDs arranged in a 2 × 5 spatial layout.

The wind turbines are equipped with LEDs for aviation obstruction marking and warning purposes. The offshore environment was developed using Unreal Engine, and the turbine models were based on assets provided by Epic Games. These models allow customization of parameters such as height, rotor diameter, rotational speed, and direction, enabling realistic simulation of offshore wind farm conditions. Figure 3 provides an overview of the virtual offshore wind farm used in this study, which consists of ten turbines equipped with aviation warning LEDs arranged in a 2 × 5 spatial configuration. The visualization was captured from a virtual camera positioned at (0, 0, 12,000 cm) to provide an elevated view of the turbine distribution.

**Figure 4:**
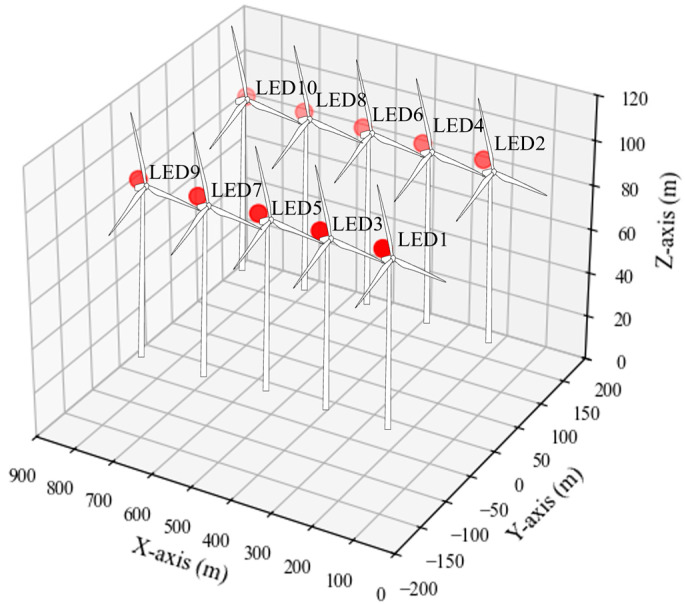
Three-dimensional LED layout in the virtual offshore wind farm environment.

Table 1 presents the predetermined absolute 3D coordinates (x, y, z) assigned to each wind turbine. Table 2 summarizes the default configuration of the virtual offshore wind farm environment. The spacing between adjacent turbines is consistently set to 150 m along the *x*-axis and 200 m along the *y*-axis, and this configuration is applied uniformly throughout the experiment. Each turbine has a blade length of 60 m and a tower height of 100 m. Figure 4 shows the 3D spatial layout of the LEDs used in the virtual offshore wind farm environment. The LED positions in the figure correspond to the geometry of the warning lights installed on the virtual turbines and reflect the turbine distribution parameters described in Table 1 and Table 2.

## 4. Proposed OCC Scheme

**Figure 5:**
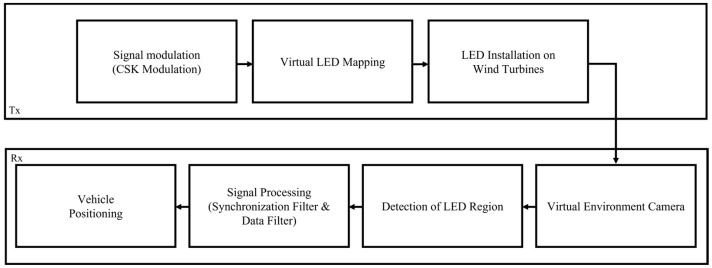
Block diagram of OCC transmitter and receiver.

Based on the virtual environment, Figure 5 presents a block diagram of the transmission and reception procedures of the OCC system.

On the transmission side, signals are assigned to LEDs through a modulation and mapping process based on CSK. The optical signals are transmitted via LEDs installed on the wind turbines. A virtual camera in the simulation environment receives these transmitted signals. In the captured images, regions of interest are detected to identify the presence of LEDs. The detected LEDs undergo signal processing to distinguish their unique identification (ID). Using this information, the PnP algorithm is applied to estimate the UAV’s 3D positions.

### 4.1. OCC Transmitter

This study employs CSK [19] for data transmission. CSK was first defined in the physical layer (PHY III) of the IEEE 802.15.7 standard. It is a high-speed modulation technique that uses multicolored LEDs to encode bit patterns as color combinations, representing various symbols [19]. On the receiver side, the color information captured by the image sensor is analyzed in a color coordinate system to decode symbols based on the detected color combinations.

According to the “Installation and Management Standards for Aviation Obstruction Lights and Daytime Markers” issued by the Ministry of Land, Infrastructure, and Transport (MOLIT) [20], medium-intensity LED-based aviation lights are mandatory on wind turbines to ensure aviation safety and nighttime visibility. These lights must remain continuously illuminated for 24 h. This study adopts a CSK modulation method that leverages the always-on LED infrastructure and applies LED color modulation for communication. MOLIT guidelines [20] specify allowable LED colors for wind turbines as red, green, blue, yellow, and white. In this study, red, green, blue, and yellow were used as modulation colors, and white was replaced with magenta to improve symbol recognition performance.

To validate this selection, an experiment was conducted with the UAV positioned at a distance of approximately 300 m from the LED source. The magenta LED achieved a detection rate of 99.44%, whereas the white LED achieved only 17.83% under the same conditions. These results demonstrate that magenta provides significantly higher detection reliability compared to white. The LEDs were implemented to transmit signals according to the color patterns defined in Table 3. Considering the practical LED specifications, the transmission rate was set to 1 bps. The adoption of CSK in this study is motivated by the operational and regulatory constraints of real offshore wind turbine environments. Since aviation obstruction lights must remain continuously illuminated and are limited to extremely low blinking frequencies [20], intensity-based modulation methods such as OOK or PWM cannot be applied. Moreover, fixed-color beacon approaches do not scale well to large wind farms where multiple turbines must be uniquely identified. CSK enables information transmission through color transitions while maintaining continuous illumination, offering both regulatory applicability and scalability for multi-turbine deployment. Therefore, CSK represents a practical and feasible modulation strategy for real offshore applications.

The data frame comprises three bits: one synchronization bit and two information bits. The synchronization bit is fixed as red, whereas the information bits are modulated according to predefined color patterns. In this context, sync data corresponds to the synchronization bit, info data1 to the first information bit, and info data2 to the second information bit. The absolute coordinates of each wind turbine are transmitted and received using these unique LED color patterns (Table 3).

### 4.2. OCC Receiver

In a V2I environment, the UAV-mounted camera captures images of the offshore wind farm, enabling identification of each wind turbine based on information transmitted from its installed LEDs. Each turbine is identified by detecting and analyzing the regions corresponding to its LEDs in the captured images. The camera then extracts the 3D absolute coordinates of the turbine and the 2D pixel coordinates of the LED. This information is subsequently used to estimate the UAV’s position. In the virtual offshore wind farm implemented in Unreal Engine, the camera moved along the *x*-axis from 0 to 50,000 cm in 2000 cm increments, corresponding to 0 to 50,000 Unreal Engine units (1 unit = 1 cm). Unreal Engine uses centimeters as its default coordinate system [21], and all experiments followed this unit convention. Table 4 summarizes the camera specifications.

Each captured image was processed to detect LEDs on the wind turbines using a You Only Look Once Version 8 (YOLOv8)-based object detection model. YOLOv8, released by Ultralytics [22], offers higher accuracy and speed compared to previous YOLO versions and incorporates new architectural features and optimizations for real-time object detection tasks [23]. The YOLOv8 architecture balances real-time performance with high precision by replacing the anchor-based detection approach and coupled head structure of YOLOv5 with an anchor-free architecture and decoupled head design. The decoupled head separates classification and regression tasks, improving training stability and detection accuracy. For this study, the YOLOv8-n (nano) model, the smallest and most lightweight variant, was adopted to enable practical deployment on computationally constrained UAV platforms. The model comprises 225 layers with 3,011,043 parameters, requiring 8.2 GFLOPs, and has a compact file size of approximately 6 MB. Using a 1920 × 1080 input resolution, it achieved 61.02 FPS on an NVIDIA RTX 3060 GPU and 6.91 FPS on an Intel Core i7 CPU. In the proposed OCC scenario, turbine LEDs operate at a 1 Hz cycle, and CSK-based color modulation requires capturing approximately 50–70 frames within 3 s to support reliable demodulation. While the baseline CPU performance of 6.91 FPS suffices for simple ON/OFF detection, it is inadequate for CSK symbol extraction due to insufficient temporal sampling. To address this, a detection-interval strategy was implemented, performing YOLO inference once every 10 frames while maintaining continuous LED tracking in the intermediate frames. This approach increased effective processing throughput to 54.51 FPS, enabling the acquisition of approximately 163 frames within 3 s, fully satisfying the temporal resolution requirements for CSK demodulation. These results demonstrate that the proposed LED detection pipeline can operate in real time even under CPU-only conditions, without requiring GPU resources. Table 5 summarizes the YOLOv8-n model specifications and inference performance, while Table 6 lists the training hyperparameters used during model optimization.

**Figure 6:**
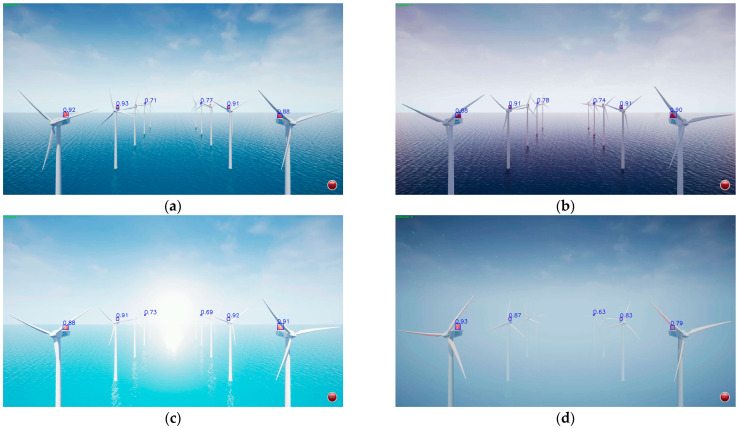
Examples of LED detection results using YOLOv8-n across four environmental conditions. (**a**) Daylight; (**b**) Evening; (**c**) Sunlight Glare; (**d**) Fog and Rain.

Bounding boxes around the wind turbine LEDs were detected using YOLOv8, and the center point of each bounding box was used to track color changes over time, enabling analysis of the unique color patterns associated with each LED. This process identifies each LED’s unique ID, its 3D absolute coordinates, and the corresponding 2D pixel coordinates in the image. Once at least four LEDs are identified, the PnP algorithm is applied to estimate the UAV’s position. When multiple LEDs are detected, the four closest LEDs are selected for localization, as detection accuracy and resolution degrade with distance. LEDs nearer to the UAV (i.e., lower in the image) are prioritized because they provide more reliable recognition. To ensure stable LED recognition under realistic offshore wind farm conditions, a confidence-based filtering strategy was applied to YOLOv8 detection results. Each detected LED is associated with a confidence score, and only detections with confidence values above 0.5 were retained. This approach suppresses unreliable bounding boxes caused by sunlight glare, sea-surface reflections, camera vibration, motion blur, and changes in exposure and white balance. The YOLOv8-n model demonstrated strong robustness across four environmental scenarios, daylight, low-light, specular reflection, and weather variation, achieving a precision of 1.000 with no false positives. Recall varied depending on the conditions; however, the frame-level detection rate remained 100% in all cases, enabling continuous LED tracking for CSK-based symbol extraction and reliable PnP-based UAV localization. These results verify that the YOLOv8-n-based LED detection approach provides significantly higher robustness and ID-tracking stability compared to traditional thresholding or blob-based segmentation methods, particularly under rapid lighting fluctuations or partial LED occlusions. Accordingly, this method ensures dependable acquisition of LED positional and color information, forming a reliable foundation for optical communication decoding and UAV navigation in real offshore wind power environments. All experiments were conducted within a virtual offshore wind farm environment, with the camera viewpoint fixed at the global coordinate (0, 0, 12,000) and a ground truth LED count of 10. The LED spatial configuration and camera position were held constant, while environmental conditions varied, including changes in illumination, strong specular reflection, weather variations such as fog and rain, and low-light conditions. This controlled setup enabled a rigorous evaluation of the YOLOv8-n detection approach under diverse visual disturbances. Representative detection examples for the four environmental conditions are illustrated in Figure 6, demonstrating the visual characteristics of each scenario. Performance comparison results are summarized in Table 7, quantitatively evaluating the model’s detection stability under varying illumination and weather conditions. These results confirm that the proposed YOLOv8-n detection approach maintains stable performance and prevents false-positive detections, even under severe environmental variations.

**Figure 7:**
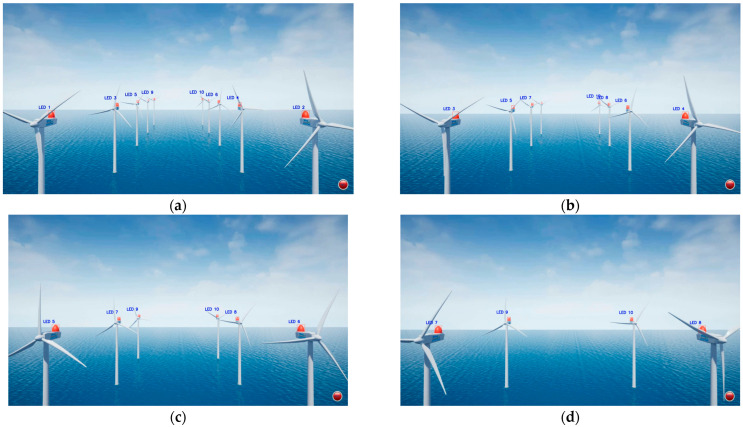
Example LED detection and identification results at different UAV positions in the virtual offshore wind farm environment: (**a**) UAV at (0, 0, 12,000 cm), (**b**) UAV at (16,000, 0, 12,000 cm), (**c**) UAV at (32,000, 0, 12,000 cm), (**d**) UAV at (48,000, 0, 12,000 cm).

Figure 7 presents example results of LED detection and identification at different UAV positions in the virtual offshore wind farm environment. The UAV captured video at 24 fps while moving along the *x*-axis. LED recognition was performed using CSK-based color decoding, where signal processing begins when the first detected frame registers red as the synchronization bit, followed by a three-bit color pattern sequence for LED ID assignment. As the distance between the UAV and the wind turbines increased, the number of LEDs that could be reliably detected and classified gradually decreased due to reduced visibility and resolution loss at longer ranges. In Figure 7a, with the UAV positioned at (0, 0, 12,000), eight of ten LEDs were successfully identified. In Figure 7b, at position (16,000, 0, 12,000), seven of eight LEDs were correctly recognized. In Figure 7c, captured from (32,000, 0, 12,000), six LEDs were accurately detected. Finally, in Figure 7d, taken from (48,000, 0, 12,000), all four LEDs were successfully identified. These results confirm that both the number of detected LEDs and recognition accuracy depend on the UAV-to-turbine distance. Even with a high-resolution camera, resolution loss and reduced detection rates occur at longer distances. Therefore, the four nearest LEDs with the highest detection confidence were prioritized to ensure stable and accurate PnP-based UAV localization.

To ensure stable decoding of CSK-based color symbols and reliable LED identification under realistic offshore conditions, temporal and spatial stabilization strategies were integrated into the decoding process. After detecting the initial LED bounding boxes with YOLOv8-n, the center coordinates were fixed as regions of interest (ROIs). Subsequent frames sampled only the pixel values within these ROIs, while YOLO inference was performed every 10 frames. Only detections with confidence scores ≥0.5 were accepted, preventing incorrect bounding-box updates caused by glare, turbine-blade occlusion, or weather-induced noise. This interval-based tracking reduces unnecessary computation and prevents unstable bounding-box updates due to turbine-blade occlusion, strong sunlight glare, or camera vibration. Considering the 1 Hz CSK symbol rate and the 24 fps camera configuration, approximately 24 color samples are collected per symbol. The final symbol color is determined using Temporal Majority Voting, effectively mitigating transient color errors caused by motion blur or illumination changes. Furthermore, when LEDs overlap or are partially occluded, spatial separation based on x–y ordering and bounding-box height is applied to maintain consistent LED ID assignment across frames. This tracking-by-detection approach ensures reliable frame-to-frame ID consistency and strengthens the robustness of subsequent PnP-based UAV localization.

### 4.3. Vehicle Positioning

**Figure 8:**
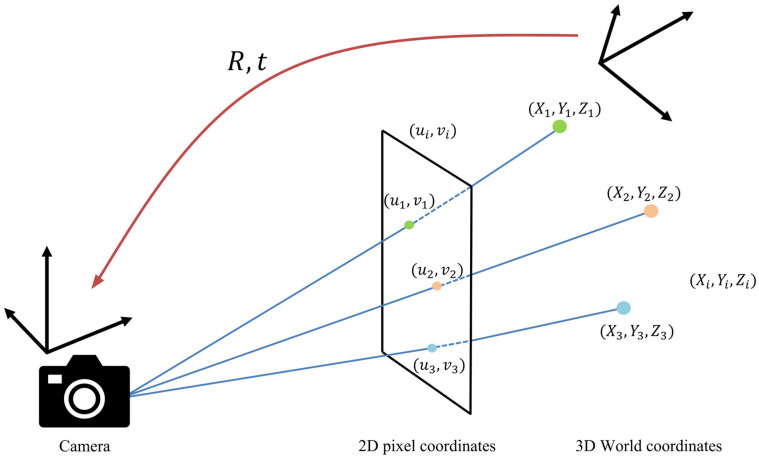
Schematic of the PnP algorithm.

Based on the reliably decoded LED IDs obtained through the proposed stabilization strategies, UAV localization was performed using the PnP algorithm. This algorithm estimates the 3D camera position by establishing correspondences between the detected 2D LED coordinates in the image plane and their known 3D coordinates in the world. For accurate operation, at least three detected LEDs must be noncollinear in 3D space and ideally well distributed across spatial locations [13]. The PnP algorithm follows the projection model:

(1) $$s\cdot \left[\begin{array}{c} u\\ v\\ 1 \end{array}\right]=K\cdot \;\left(R\cdot \left[\begin{array}{c} x\\ y\\ z \end{array}\right]+t\right),$$

where (u,v) represents the 2D pixel coordinates on the image plane, (x,y,z) denotes the 3D world coordinates, K is the camera intrinsic matrix, R is the rotation matrix, and t is the translation vector, collectively defining the camera’s position and orientation in the world coordinate frame. Figure 8 illustrates this projection relationship.

**Figure 9:**
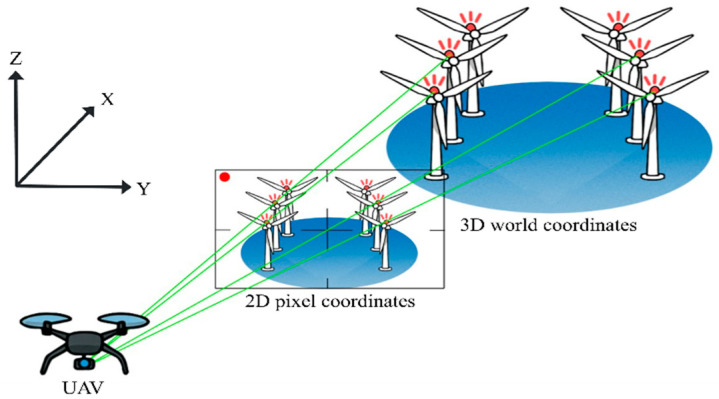
Schematic of the PnP algorithm for UAV position estimation.

In this study, the UAV position was estimated using 2D–3D correspondences from four LEDs to enhance localization accuracy and safety. Figure 9 depicts the 2D–3D point-matching process required for applying the PnP algorithm in an offshore wind farm environment. The 2D coordinates correspond to pixel locations detected in the images, while the 3D coordinates represent the absolute spatial positions defined in the Unreal Engine-based virtual environment.

The OpenCV solvePnP() function was employed to estimate the translation vector using the cv2.SOLVEPNP_ITERATIVE method, which applies Levenberg–Marquardt optimization for iterative refinement. This approach was preferred over the EPnP algorithm [24], which provides a linear O(n) solution, because it offers higher numerical accuracy with a limited number of detected LED correspondences. While EPnP is computationally efficient and suitable for large-scale feature-based applications, its linear approximation can reduce precision when only a few correspondences are available. In contrast, the iterative method continuously refines the pose estimation through nonlinear optimization, yielding more stable and accurate localization in environments with sparse, fixed-position LEDs, such as the virtual offshore wind farm scenario in this study. As an early-stage research investigation into UAV localization using OCC, this study prioritized high positional accuracy over computational speed to establish a reliable foundation for future development.

During the PnP-based localization process, errors were observed in estimating the UAV altitude (*z*-axis coordinate). This issue arises from the structural characteristics of offshore wind farms and UAV operational constraints. In industrial settings, turbines are typically designed with a constant hub height to improve power generation prediction, standardize design and manufacturing, and enhance maintenance efficiency [25,26,27]. Consequently, wind turbines display minimal variation along the *z*-axis, and UAVs must maintain a safe distance from the turbines, limiting the geometric diversity necessary for accurate altitude estimation. Although the PnP algorithm estimates UAV position by matching 2D image coordinates with corresponding 3D world coordinates, the uniform hub height results in a flat *z*-axis distribution. As a result, geometric information is insufficient for reliable altitude estimation, and localization accuracy along the *z*-axis is constrained.

This study proposes an empirical image-based *z*-axis correction algorithm that leverages indicators derived from detected LEDs to address altitude estimation limitations. The approach is based on the observation that a consistent correlation exists between the bounding box height ($h_{bbox}$) and the y-coordinate of the bounding box center ($y_{ref}$) when an LED is detected, which corresponds to the vertical distance between the UAV and the LED. As the UAV approaches the LED, $h_{bbox}$ increases, making it a useful indicator of the absolute *z*-axis distance.

In contrast, $y_{ref}$ represents the vertical position of the LED in the image frame. A higher UAV altitude relative to the LED results in the LED appearing lower in the image, making $y_{ref}$ indicative of the relative altitude difference between the UAV and the turbine. Together, these two variables provide indirect but reliable information about the *z*-axis distance and relative altitude. Without complex modeling, they allow derivation of an empirical correction coefficient $k_{z}$ to compensate for *z*-axis estimation errors:

(2) $$k_{z}=f\left(h_{bbox},y_{ref}\right).$$

The correction algorithm is implemented as a lookup table based on pre-collected simulation data. First, the image is segmented into vertical intervals according to the bounding box height $h_{bbox}$ of the detected LED. Then, based on the interval of the reference y-coordinate $y_{ref}$, an appropriate correction coefficient $k_{z}$ is selected. This coefficient is multiplied by the initial z-coordinate estimated using the standard PnP algorithm to generate the corrected z-coordinate:

(3) $$Z_{corrected}=Z_{pnp}\cdot k_{z}$$

In the workflow, among the detected LEDs in the image, the four highest-priority LEDs are selected. The two closest to the camera (foreground) are designated as reference points. The average of their bounding box heights and y-coordinates is computed, and the corresponding correction coefficient is applied to the PnP-estimated z-coordinate to produce the final corrected position.

Given the repetitive nature of UAV patrol operations in offshore wind farms, if the camera configuration remains fixed $k_{z}$ can be determined during an initial flight and reused without additional calibration. In this study, $k_{z}$ was derived within a digital-twin-based virtual offshore wind farm created in Unreal Engine. The UAV flight altitude (12,000 cm), camera focal length (12 mm), LED installation height (10,250 cm), and a 2 × 5 turbine grid layout were modeled according to the experimental scenario. The coefficient derived under these conditions can be consistently applied within the same operational environment without recalibration.

Table 8 presents the *z*-axis correction coefficients in a lookup table format, organized by bounding box height and vertical pixel position based on pre-collected simulation data. Since LED detection becomes unreliable at excessively high or low altitudes, experiments were conducted only within ranges where LEDs could be consistently captured, ensuring the validity and accuracy of the correction within the empirically defined effective region. Although $k_{z}$ is empirically derived and specific to the modeled environment, the algorithm can be generalized by constructing a digital twin of a real offshore wind farm and extracting a site-specific coefficient using the same procedure. This approach enables practical scalability to new operational sites without repeated physical measurements or additional model training and provides a feasible pathway for real-world deployment of the UAV-based localization framework.

## 5. Experimental Results

**Figure 10:**
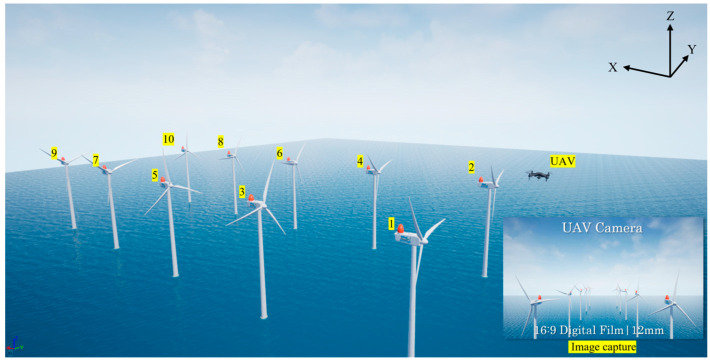
Experimental Environment.

The experiment was designed by capturing images at fixed intervals along a predefined UAV flight path to evaluate UAV localization in a virtual simulation environment based on Unreal Engine. Figure 10 presents the simulation environment, where the UAV moves along the *x*-axis from 0 to 50,000 cm. For the simulation, the intrinsic camera parameters provided by Unreal Engine were used without modification, and the UAV orientation was assumed fixed to focus solely on position estimation.

Snapshots were taken at 2000 cm intervals, resulting in 26 snapshots, from positions where all ten wind turbines were fully visible to positions where at least four turbines could be observed in the camera’s field of view. This procedure created a stepwise movement path for the UAV. At each position, the camera captured the wind turbines and detected the LEDs. In each frame where four or more LEDs were detected, their unique IDs were used to retrieve the absolute 3D coordinates, which were then matched with the corresponding 2D pixel coordinates detected by the image sensor. The PnP algorithm was subsequently applied to estimate the UAV position.

**Figure 11:**
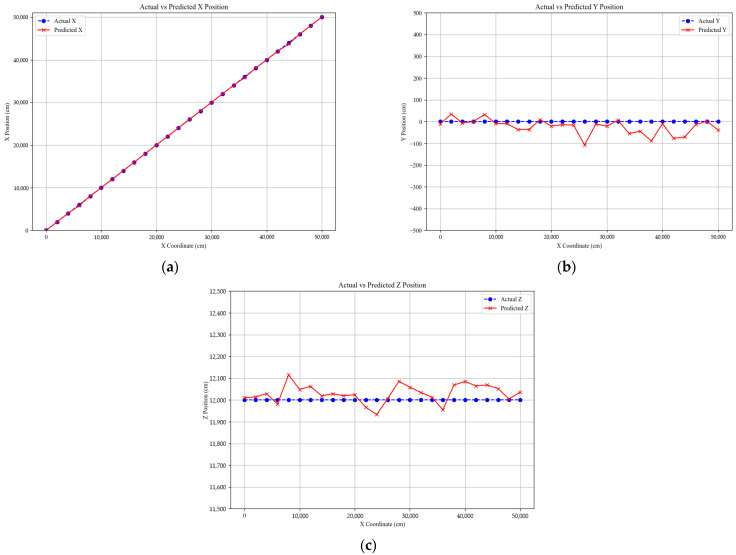
UAV position estimation results comparing actual and predicted coordinates along: (**a**) X-, (**b**) Y-, and (**c**) Z-axes.

Figure 11 visualizes the UAV localization results by comparing the actual and estimated trajectories along the x-, y-, and z-axes. In all three plots, the horizontal axis represents the actual UAV travel distance along the x-direction from 0 to 50,000 cm, while the vertical axes correspond to the estimated x-, y-, and z-coordinate values, allowing independent evaluation of localization performance along each axis. Both the ground-truth trajectory and the predicted trajectory are plotted together for direct visual comparison.

The results indicate that the estimated coordinates closely follow the actual path with relatively small deviations, demonstrating stable localization performance across the entire flight range. In Figure 11a, the estimated *x*-axis trajectory aligns closely with the true path. In Figure 11b, the estimated y-coordinates remain primarily within ±50 cm, reflecting stable lateral accuracy. In Figure 11c, the estimated z-coordinates maintain consistent altitude, remaining within approximately ±100 cm despite variations in distance.

To provide quantitative verification, Table 9 presents the mean absolute error (MAE) and mean error (ME) for each coordinate axis under fixed-altitude conditions. The MAE was approximately 40.3 cm for the *x*-axis, 30.0 cm for the *y*-axis, and 43.0 cm for the *z*-axis, with corresponding ME values of −24.4, −23.5, and +30.4 cm. Overall, the localization error remained within ±45 cm across all axes, confirming stable and uniform positioning accuracy under constant flight height. The small differences in error magnitude among the three axes indicate that the proposed PnP-based localization framework achieves balanced performance in both horizontal and vertical directions when altitude variation is limited.

**Figure 12:**
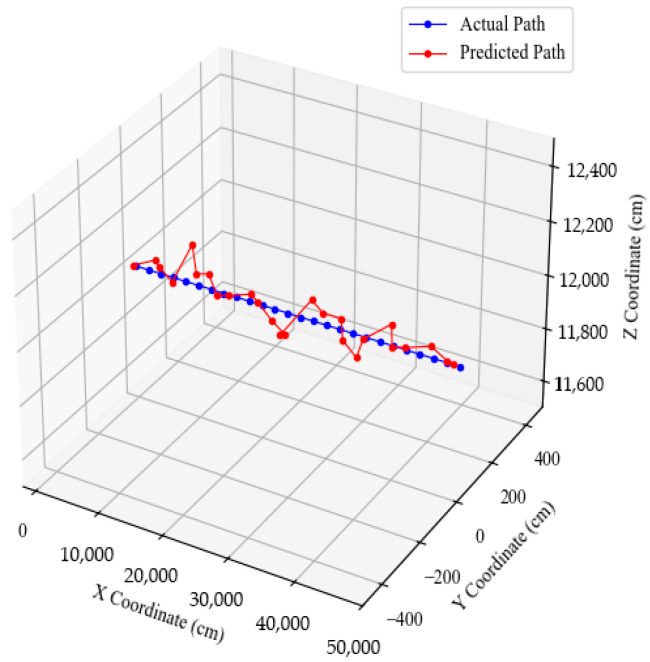
UAV position estimation results in 3D trajectories.

Figure 12 presents a 3D trajectory comparison graph, visualizing the actual and estimated UAV flight paths. The two trajectories closely align throughout the flight, confirming overall geometric similarity and consistent localization performance of the proposed method. The estimated trajectory successfully follows the curvature and altitude transitions of the actual path, demonstrating smooth tracking without abrupt deviations. This visual agreement further validates the reliability of the PnP-based localization framework in reconstructing UAV motion in 3D space.

Based on these experimental results, the proposed localization system demonstrated high accuracy in the virtual environment, achieving submeter precision in most cases along a 500 m UAV flight path. Although minor localized deviations were observed, the average localization error remained below 50 cm, confirming the robustness of the system. These findings indicate that accurate real-time localization can be achieved using only LEDs and a camera, without additional external sensors. This demonstrates the potential of the system for effective operation in environments where GPS is restricted or unavailable and confirms the feasibility of stable UAV localization for O&M tasks in constrained environments, such as offshore wind farms.

Additional experiments were conducted by varying the UAV flight altitude to further evaluate the performance of the proposed *z*-axis correction algorithm. In this experiment, the UAV followed an *x*-axis flight path from 0 to 40,000 cm while the altitude gradually increased from approximately 12,000 to 16,000 cm. At each altitude, the UAV captured images of the LEDs installed on the wind turbines and estimated its 3D position using the PnP algorithm combined with the ^z^-axis correction method. This procedure allowed assessment of localization performance under continuously varying altitude conditions, simulating realistic flight scenarios with multiple height transitions.

**Figure 13:**
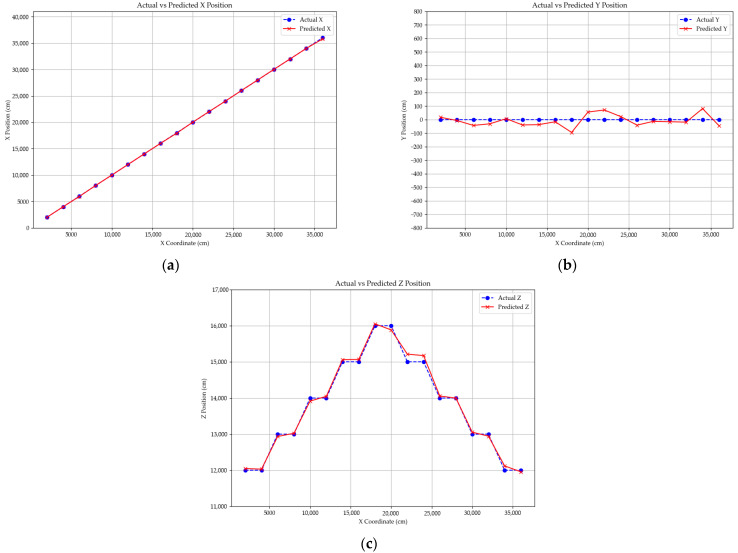
UAV position estimation results under altitude variation, comparing actual and predicted coordinates along: (**a**) X-, (**b**) Y-, and (**c**) Z-axes.

Figure 13 visualizes the UAV localization results under varying altitude conditions by comparing the actual and estimated trajectories along the x-, y-, and z-axes. In all plots, the horizontal axis represents the actual UAV movement along the x-direction from 0 to 50,000 cm, while the vertical axes correspond to the estimated x-, y-, and z-coordinate values, enabling independent evaluation of localization performance under changing altitude conditions. Both the ground-truth and predicted trajectories are plotted together for direct visual comparison.

The results indicate that the predicted coordinates closely follow the actual flight path despite altitude variations, demonstrating reliable localization performance. In Figure 13a, the estimated ^x^-axis trajectory aligns well with the true path, exhibiting only minor deviations. In Figure 13b, the estimated y-coordinates remain within a narrow band around the actual values, showing stable lateral accuracy throughout the flight. In Figure 13c, even as the altitude gradually changes, the estimated z-coordinates smoothly track the actual altitude profile, confirming the effectiveness of the proposed ^z^-axis correction model.

To provide quantitative verification of these visual observations, Table 10 presents the MAE and ME for each axis under varying altitude conditions. The MAE was approximately 29.2 cm for the *x*-axis, 36.3 cm for the *y*-axis, and 72.6 cm for the *z*-axis, with corresponding ME values of −10.8 cm, −7.9 cm, and +31.4 cm, respectively. The *z*-axis exhibited the largest error, primarily because its estimation relies solely on image-based geometric information between the camera and the LEDs, without direct depth sensing. Additionally, the *z*-axis correction coefficient was empirically derived from a limited set of simulation data rather than external range-measuring sensors. As further empirical data are collected under diverse lighting, distance, and motion conditions, the robustness of this coefficient is expected to improve, enhancing the accuracy and stability of altitude estimation.

**Figure 14:**
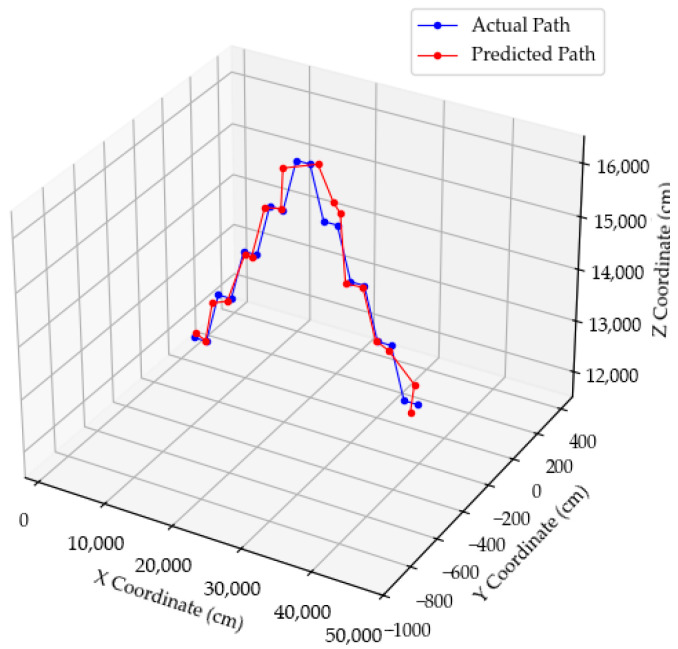
UAV position estimation results in 3D trajectories during altitude variation.

Figure 14 presents a 3D comparison of the actual and estimated UAV flight trajectories. The two trajectories exhibit a high degree of geometric consistency, maintaining nearly identical curvature and slope transitions throughout the flight. Despite changes in flight altitude, the estimated trajectory continuously follows the actual path, demonstrating the stability and reliability of the proposed localization method under varying altitude conditions. Minor vertical deviations appear near altitude-transition points, primarily due to the limited resolution of the *z*-axis correction coefficient and the absence of direct depth sensing. Nevertheless, the overall alignment confirms that the integrated framework maintains smooth tracking and stable positional updates without cumulative drift, ensuring robust 3D localization performance suitable for real UAV operations, such as offshore inspection and wind farm maintenance.

These experimental results demonstrate that the proposed *z*-axis correction algorithm functions effectively, enabling stable localization even when UAV altitude continuously changes. The estimated UAV positions closely match the actual trajectory, maintaining submeter accuracy at most points. This finding is significant for offshore wind farm applications, where LEDs attached to turbines are typically positioned uniformly along the *z*-axis at fixed altitudes. Such structural uniformity can amplify localization errors when the UAV altitude varies. The proposed correction algorithm mitigates these constraints, providing robust estimation in complex 3D environments. It also exhibits strong generalization performance, maintaining accurate localization under varying altitude conditions.

## 6. Conclusions

This study proposed an OCC-based UAV positioning framework for offshore wind farm environments and conducted experimental validation within a digital twin environment implemented using Unreal Engine. In the proposed system, aviation obstruction lights (LEDs) mounted on wind turbines serve as transmitters, while a UAV-mounted camera functions as the receiver, enabling 3D position estimation without relying on GNSS or additional RF sensors. LED signals are transmitted using CSK modulation and processed through a YOLOv8-n-based LED detection model integrated with the PnP algorithm for real-time positioning. To ensure reliable LED identification and stable CSK decoding under realistic offshore conditions, temporal and spatial stabilization strategies were applied. Temporal Majority Voting aggregated multiple color samples per symbol, mitigating transient decoding errors caused by motion blur or illumination variation. Spatial separation and tracking-by-detection strategies preserved consistent LED ID assignment in cases of overlapping or partially occluded LEDs. These stabilization mechanisms enhanced LED tracking robustness and strengthened the reliability of subsequent PnP-based UAV localization. To address altitude estimation instability arising from the vertically uniform distribution of LEDs on offshore wind turbines, an empirical z-axis correction method based on image-derived features was implemented. Experimental results demonstrated that the proposed framework achieved an MAE of less than one meter across all axes, with the estimated UAV trajectory closely matching the ground-truth flight path. The z-axis correction effectively mitigated shallow-angle depth ambiguities and maintained stable altitude estimation under varying flight heights. These findings indicate that OCC-based UAV positioning is a feasible alternative in GNSS-denied or interference-prone environments. However, the evaluation was limited to simulation-based experiments. Although environmental factors such as illumination variation, sea-surface reflections, vibration, motion blur, and LED brightness constraints were considered during LED detection and decoding robustness tests, they were not fully incorporated into the PnP-based localization validation. Future work will involve field verification in real offshore wind farm environments, adaptive *z*-axis compensation using real-world data, and multi-LED fusion techniques to enhance robustness and practical applicability. In summary, this study provides preliminary evidence supporting the feasibility of OCC-based UAV positioning and lays a foundation for UAV-based autonomous O&M activities in offshore wind farm environments.

## Figures and Tables

**Table 1:** Predetermined absolute coordinate data of offshore wind power generators.

Wind Turbine LED (i)	x (cm)	y (cm)	z (cm)
Wind turbine LED (1)	16,000	−10,000	10,250
Wind turbine LED (2)	16,000	10,000	10,250
Wind turbine LED (3)	31,000	−10,000	10,250
Wind turbine LED (4)	31,000	10,000	10,250
Wind turbine LED (5)	46,000	−10,000	10,250
Wind turbine LED (6)	46,000	10,000	10,250
Wind turbine LED (7)	61,000	−10,000	10,250
Wind turbine LED (8)	61,000	10,000	10,250
Wind turbine LED (9)	76,000	−10,000	10,250
Wind turbine LED (10)	76,000	10,000	10,250

**Table 2:** Basic setup environment for offshore wind farm.

Category	Parameter	Value
Wind farm layout	Number of wind turbines	10 (2 × 5 grid)
	Distance between turbines	150 m (*X*-axis), 20 m (*Y*-axis)
Wind turbine specifications	Tower height	100 m
	Blade length	60 m

**Table 3:** Predetermined color patterns assigned to offshore wind turbines.

Wind Turbine LED (i)	Sync Data	Info Data 1	Info Data 2
Wind turbine LED (1)	Red	Green	Magenta
Wind turbine LED (2)	Red	Magenta	Green
Wind turbine LED (3)	Red	Green	Yellow
Wind turbine LED (4)	Red	Yellow	Green
Wind turbine LED (5)	Red	Magenta	Yellow
Wind turbine LED (6)	Red	Yellow	Magenta
Wind turbine LED (7)	Red	Blue	Green
Wind turbine LED (8)	Red	Green	Blue
Wind turbine LED (9)	Red	Yellow	Blue
Wind turbine LED (10)	Red	Blue	Yellow

**Table 4:** Camera specifications in the experiment.

Parameter	Value
Frame Rate	24 FPS
Resolution	3840 × 2160 (16:9)
Sensor Width	23.76 mm
Sensor Height	13.365 mm
Lens Type	12 mm Prime Lens
Focal Length	12.0 mm (fixed)
Aperture Range	f/2.8–f/22.0

**Table 5:** YOLOv8-n model specification and inference performance.

Model Variant	YOLOv8-n
Parameters	3,011,043
FLOPs	8.2 GFLOPs
Model Size	~6 MB
Input Resolution	1920 × 1080
Inference FPS (GPU)	61.02 FPS
Inference FPS (CPU)	6.91 FPS
Inference FPS (CPU, 10 Interval frames)	54.51 FPS
Real time Feasibility	Suitable for real-time UAV operation

**Table 6:** Learning Parameters.

Parameter	Value
epoch	200
train dataset	1180
validation dataset	100
optimization	Stochastic gradient descent
loss function	Binary cross entropy, focal, CloU
learning rate range	Initial ($1\times 10^{-5}$ to 0.01), Final Factor (0.01)

**Table 7:** Quantitative evaluation of YOLOv8-n-based LED detection performance under diverse offshore wind-farm environments.

Scenario	Precision	Recall	F1 Score	Avg Conf	Avg FN per Frame
Daylight (normal)	1.0000	0.6077	0.7560	0.860	3.92
Evening	1.0000	0.5857	0.7387	0.835	4.14
Sunlight Glare	1.0000	0.4561	0.6265	0.861	5.44
Fog and Rain	1.0000	0.4265	0.5979	0.852	5.75

**Table 8:** *Z*-axis Correction Lookup Table.

Bounding Box Height Range	Vertical Pixel Location Range (Pixels)	Z-Axis Correction Coefficient
h ≤ 27	540–580	1.23
	581–630	1.37
	631–660	1.67
	661–700	2.07
	701–740	2.63
	741–790	3.42
28 ≤ h ≤ 39	570–610	1.23
	611–650	1.37
	651–690	1.67
	691–730	2.07
	731–790	2.63
	791–840	3.42
40 ≤ h ≤ 50	590–630	1.23
	631–690	1.37
	691–750	1.67
	751–810	2.07
	811–870	2.63
	871–950	3.42
51 ≤ h	600–660	1.23
	661–730	1.37
	731–800	1.67
	801–860	2.07
	861–950	2.63
	951–1030	3.42

**Table 9:** Mean absolute error and mean error.

	x (cm)	y (cm)	z (cm)
Mean absolute error (MAE)	40.29	30.02	42.96
Mean error (ME)	−24.41	−23.52	30.42

**Table 10:** Mean absolute error and mean error for position estimation under changing altitude conditions by axis.

	x (cm)	y (cm)	z (cm)
Mean absolute error (MAE)	29.16	36.32	72.56
Mean error (ME)	−10.75	−7.89	31.44

## Data Availability

The data presented in this study are available in the article. Further inquiries can be directed to the corresponding author.
